# Supplementary material for: Biophysical impacts of earth greening can substantially mitigate regional land surface temperature warming
Source: Nat Commun. 2023 Jan 9;14:121. doi: 10.1038/s41467-023-35799-4 (PMC9829907; doi:10.1038/s41467-023-35799-4)
Supplement: Supplementary file 1 — Supplementary Information [file 41467_2023_35799_MOESM1_ESM.pdf]

Supplementary information for:

Biophysical Impacts of Earth Greening Can Substantially Mitigate Regional Land Surface Temperature Warming

Supplementary Discussion

1. Decomposition of LST sensitivity

The LST sensitivity could be considered as the result of the competition between non-radiative and radiative processes. Based on the energy balance equation, we decompose the LST sensitivity into the contribution from each part of the energy terms for the quantitative attribution analysis (see Methods). Notably, decomposition results have lower spatial coverage (Supplementary Fig. 3 vs. Fig. 1a), mainly due to the missing data issue of satellite observations in high-latitude winters and the coarser input data resolution. Annually, we confirm the negative but slight temperature effect from the indirect climate feedback ($\frac{dLST_{bio}^{SW\downarrow}}{dLAI}$, -0.09 K m^2 m^{-2}; $\frac{dLST_{bio}^{LW\downarrow}}{dLAI}$, 0.05 K m^2 m^{-2}) (Supplementary Fig. 3d, e), mainly due to the reduction in incoming shortwave radiation for the greener regions induced by the mesoscale cloud enhancement effect for the greener surface[1]. In line with previous studies, our results show that enhanced evapotranspiration dominates the cooling of most global areas[2], whose magnitude is far larger than the final LST sensitivity ($\frac{dLST_{bio}^{LE}}{dLAI}$, -2.14 K m^2 m^{-2}) (Supplementary Fig. 3b). Moreover, as the increasing energy diffused through water evaporation is accompanied by less heat convection[3], approximately half of evaporative cooling is offset by warming from the reduction in sensible heat ($\frac{dLST_{bio}^{H}}{dLAI}$, 1.10 K m^2 m^{-2}) (Fig. 3c). Finally, the increasing LAI leads to a decrease in albedo, thus intensifying solar radiation absorption and generating a warming effect ($\frac{dLST_{bio}^{\alpha}}{dLAI}$, 0.37 K m^2 m^{-2}) (Supplementary Fig. 3a). This albedo warming is relatively weak at the global scale, but it is dominant in boreal regions.

2. Sensitivity from different regression methods

The correct biophysical LST sensitivity is the precondition of quantifying the climate effect of earth greening. In this paper, the sensitivity is derived from the spatial comparison of LST with different LAI. Here, we further perform a temporal statistical method to solve the LST sensitivity for comparison, which uses multi-linear regression (MLR) of time series LST, LAI, and climatic data to disentangle the temperature effects of vegetation greening[4]. The intrinsic difference between our method and MLR method is the source of regression samples. Specifically, the samples in our studies are from the spatial nearby pixels (sharing a similar background climate), while samples in MLR method are from the time series observations for a given location. The feasibility of temporal regression is debatable mainly because of the changing background climate[5]. The long-term trends and fluctuations of the climate system drive both LAI and LST variation, and thus vegetation and temperature show complex two-way effects (Supplementary Fig. 16a). However, samples from our spatial regression method sharing the same background climate, which means the signal of climate natural variability or the long-term warming trend affecting vegetation growth is excluded. The spatial variability of LST samples is mainly driven by vegetation growth (LAI affecting biophysical properties) after filtering out the impact of land cover and altitude (Supplementary Fig. 16b). Hence, our method is essentially a spatial controlled experiment, but the MLR method is an observation-based statistical method.

Further comparison of annual sensitivity from our method ($\frac{dLST_{bio}}{dLAI}$) and MLR method ($\frac{\partial LST_{bio}}{\partial LAI}$) can be found in Supplementary Fig. 10. Notably, same LAI and LST datasets were used here. Compared with our result, the annual sensitivity derived from MLR shows a stronger negative signal in the southern mid-latitude, which is mainly caused by the sensitivity difference in Australia. Meanwhile, the positive sensitivity is significantly strengthened in

boreal regions in MLR result. These differences are possible because the reversed "temperature-vegetation" effect is superimposed with the "vegetation-temperature" signal of our concern. For instance, global warming could be the major cause of greening and the stronger boreal signal in MLR is more likely the reflection of temperature influencing the vegetation growth. Conversely, the larger negative sensitivity in Australia is due to the reversed "temperature limits vegetation growth" effect overlapping with the "vegetation greening induces cooling effect". However, our spatial regression method can exclude the reversed signal of large-scale warming affecting vegetation physiology and phenology, as the sensitivity is regressed from simultaneous LST and LAI observations from spatial samples sharing the same background climate (see methods).

3. Sensitivity Tests

We performed sensitivity tests for the choice of LAI datasets and the size of moving window. Specifically, we calculated the monthly sensitivity using additional GIMMS and GLOBMAP (resampled into 0.05°) LAI datasets. Here, we did not test the MODIS C6 product, due to the missing data issue in high-latitude winter for the main look-up-table method. We compared the final temperature effect but not LST sensitivity, because differences can be found among the long-term trends from different LAI products[6]. We find almost the same spatial pattern and good latitudinal consistency of greening induced temperature effect (δLST_{bio}) from the three products (Supplementary Fig.13, 14) at seasonal or annual scale. Similarly, we repeated experiments for different window sizes of 40 km, 50 km and 60 km (about 7×7, 9×9 and 11×11 pixels near the equator, respectively) using only GLASS LAI. Reduced window size means fewer samples for regression and thus may generate higher uncertainty. However, the LST sensitivity was not significantly affected by the changing window size. We find almost the same latitudinal pattern, with all the scatters near the 1:1 line. (Supplementary Fig.15). These results show our results are robust against different LAI products and window sizes.

Supplementary figures

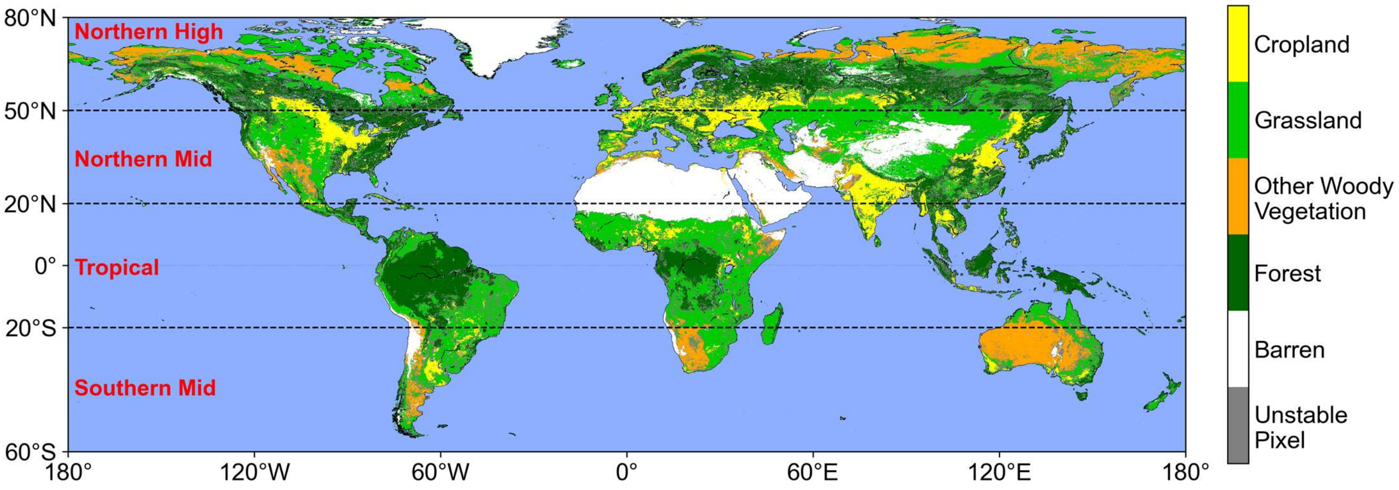

Supplementary Figure 1. **Global map of broad vegetation landcover types derived from MCD12C1 products.** Unstable pixel indicates the main landcover type has changed at least once during the study period.

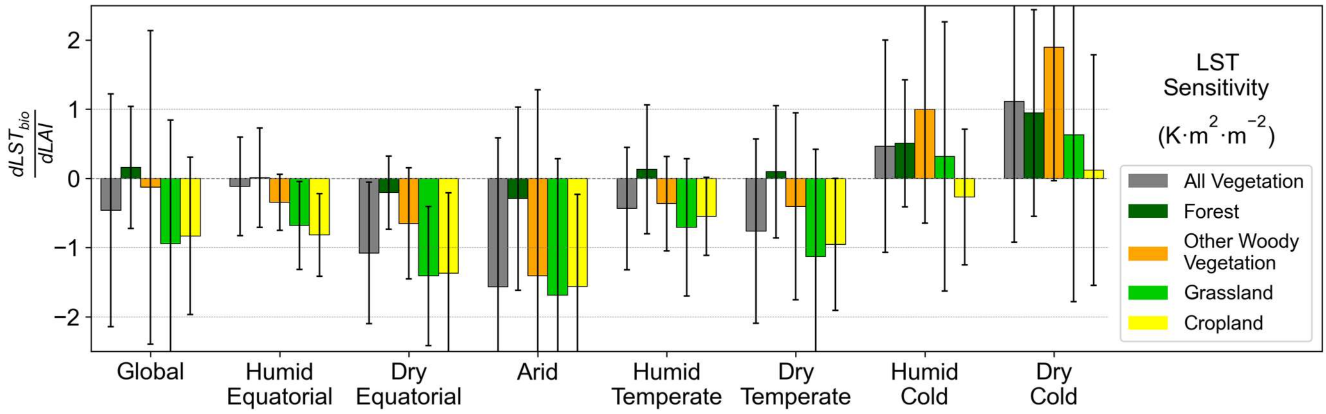

Supplementary Figure 2. **Mean sensitivity of land surface temperature to leaf area index ($\frac{dLST_{bio}}{dLAI}$) for different vegetation types and climate zones.** The error bar indicates the standard deviation. Source data are provided as a Source Data file.

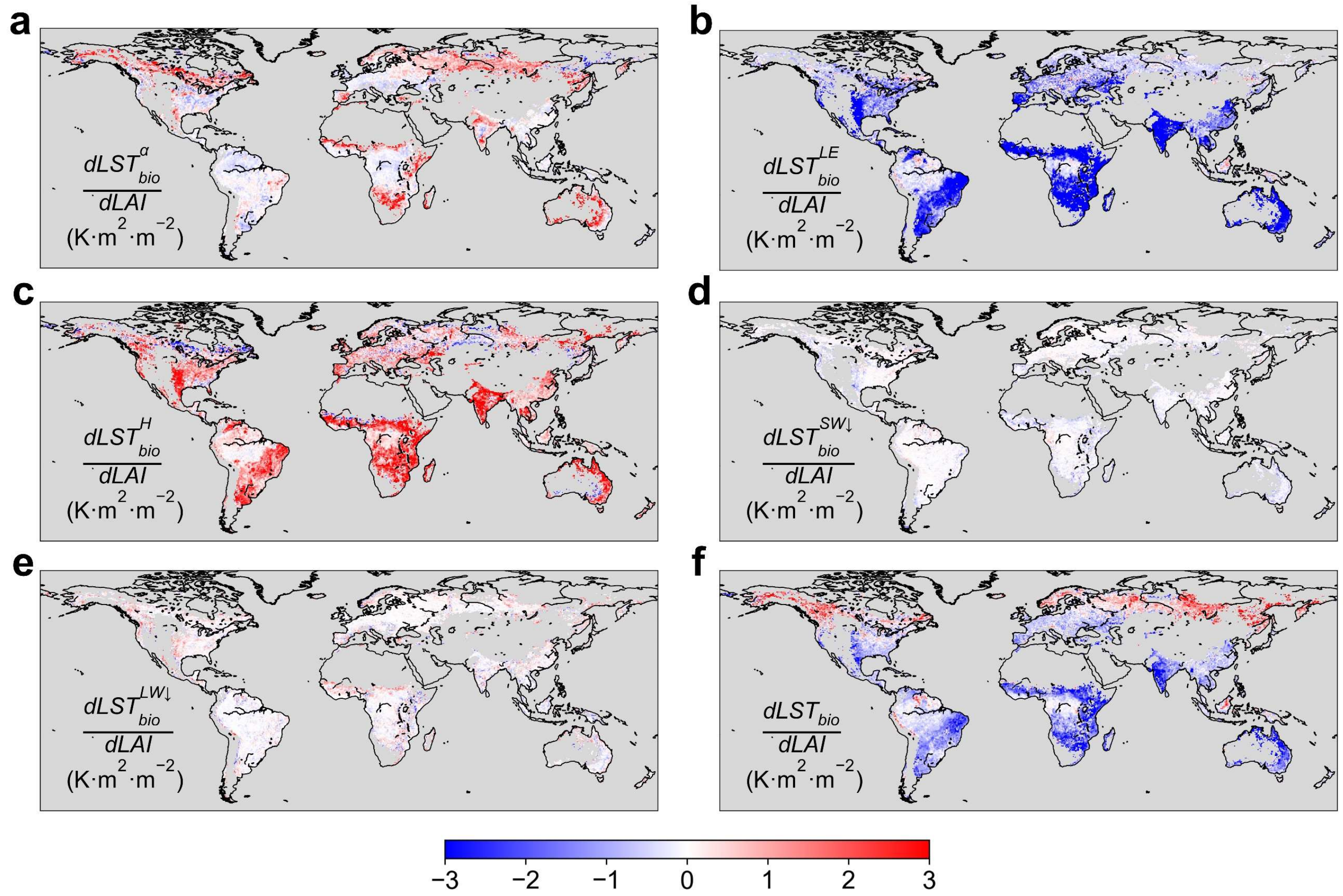

Supplementary Figure 3. **The global maps of decomposition results of annual land surface temperature (LST) sensitivity.** The global map of equivalent LST sensitivity to leaf area index (LAI) from **a** LAI-albedo ($\frac{dLST_{bio}^{\alpha}}{dLAI}$), **b** LAI-latent heat ($\frac{dLST_{bio}^{LE}}{dLAI}$), **c** LAI-sensible heat ($\frac{dLST_{bio}^{H}}{dLAI}$), **d** LAI-downward shortwave radiation ($\frac{dLST_{bio}^{SW\downarrow}}{dLAI}$), **e** LAI-downward longwave radiation ($\frac{dLST_{bio}^{LW\downarrow}}{dLAI}$) pathways, correspondingly. **f** The sum of mentioned five parts of contributions. Source data are provided as a Source Data file.

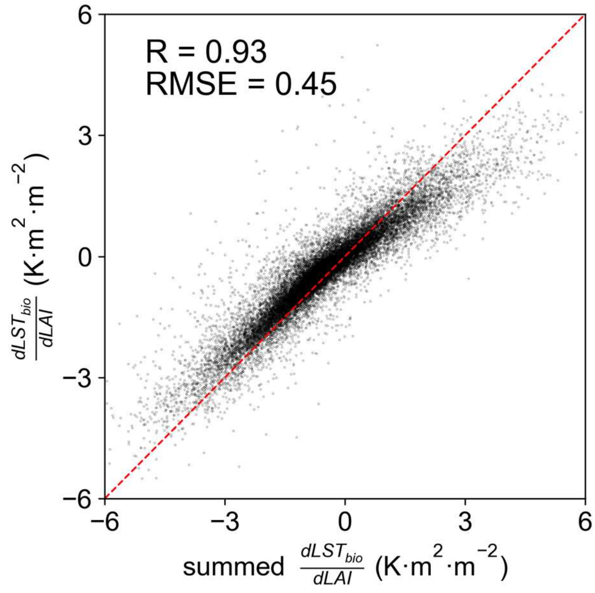

Supplementary Figure 4. **Scatter plot between the sum of decomposition sensitivities and the direct calculated land surface temperature sensitivity (Supplementary Fig. 3f vs Fig. 1a).** Source data are provided as a Source Data file.

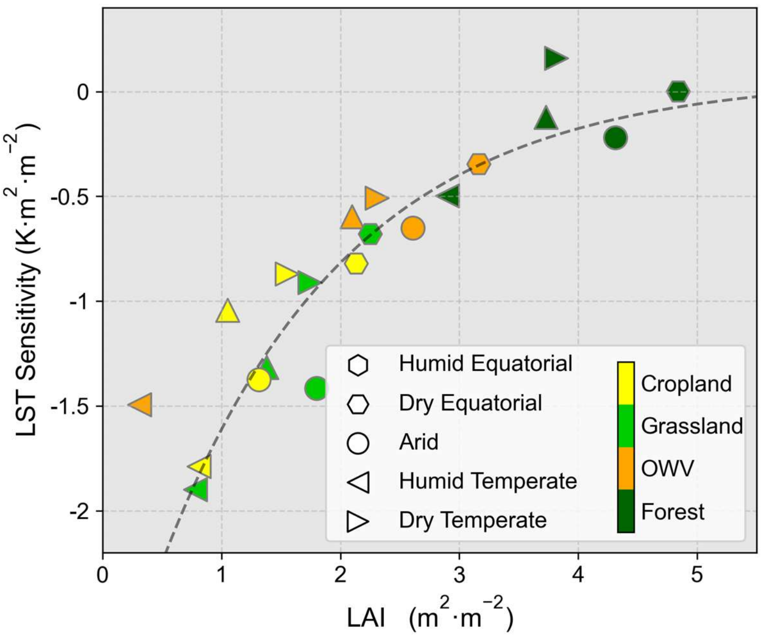

Supplementary Figure 5. **The relationship between leaf area index and annual land surface temperature sensitivity ($\frac{dLST_{bio}}{dLAI}$) at biome level.** The gray dotted line represents the exponential function in Fig.4c. Abbreviation: OWV, other vegetation. Source data are provided as a Source Data file.

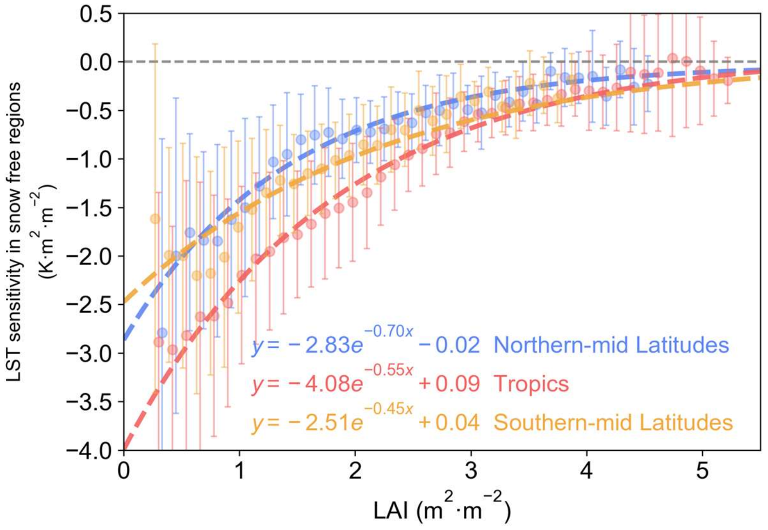

Supplementary Figure 6. **Different impacts of leaf area index variation on land surface temperature sensitivity in snow-free regions within different latitudinal zones**. Error bars show the standard error of the sensitivity within the leaf area index (LAI) bin (±0.15 m^2 m^{-2}). Source data are provided as a Source Data file.

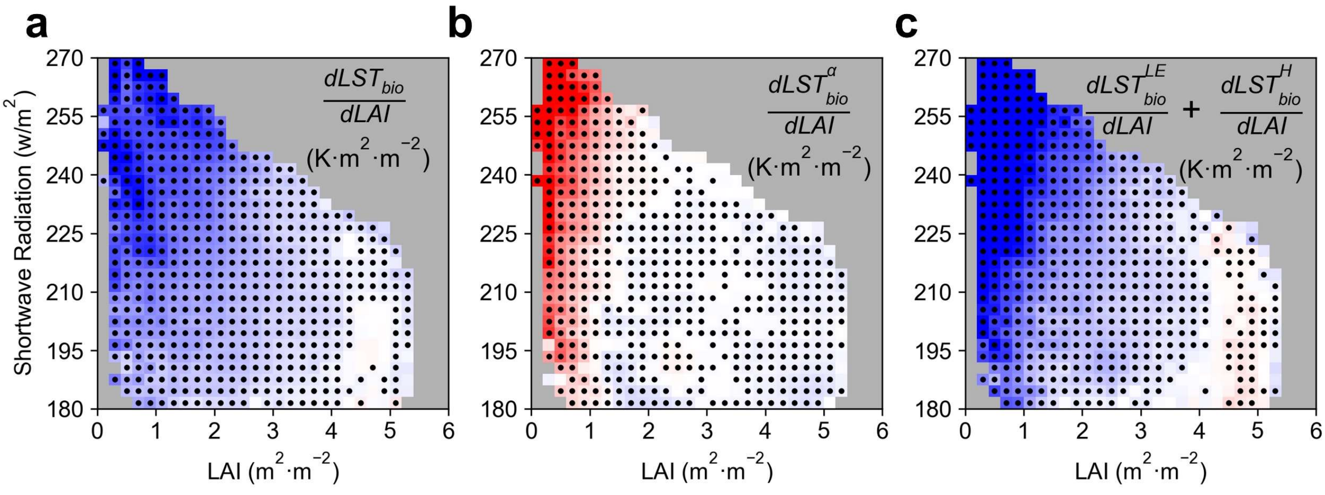

Supplementary Figure 7. **Simultaneous control of leaf area index (LAI) and downward shortwave radiation on local biophysical feedbacks in snow-free regions. a** The binned means of land surface temperature (LST) sensitivity ($\frac{dLST_{bio}}{dLAI}$) vs downward shortwave radiation and LAI. **b, c** Same as **a**, but for the equivalent LST sensitivities for the radiative process ($\frac{dLST^{\alpha}_{bio}}{dLAI}$) and non-radiative ($\frac{dLST^{LE}_{bio}}{dLAI} + \frac{dLST^{H}_{bio}}{dLAI}$) processes. Bins with black dots indicate the mean value is statistically significantly different from zero (Student's t-test; P-value < 0.05). Source data are provided as a Source Data file.

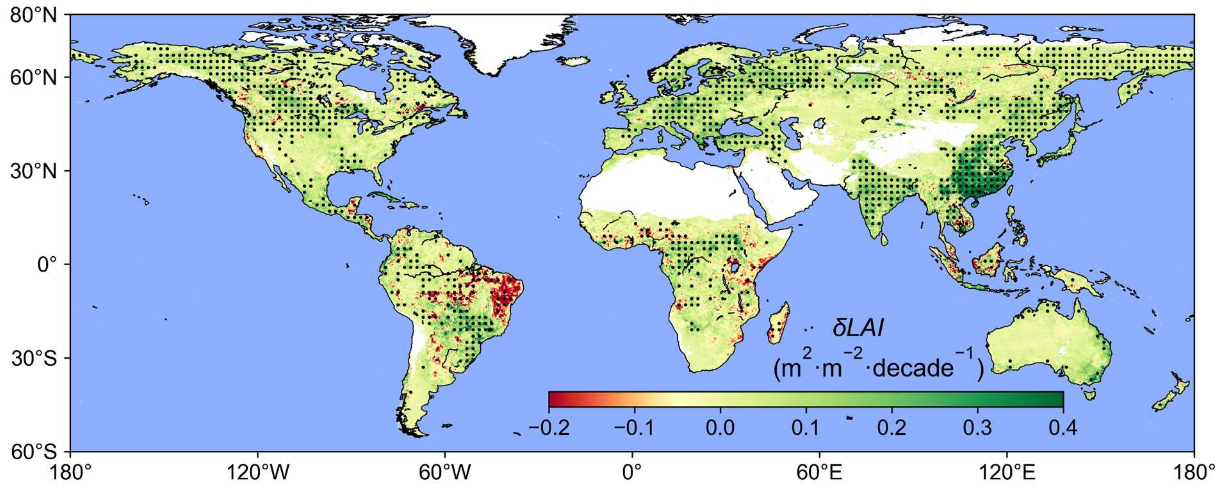

Supplementary Figure 8. **Global map of the annual leaf area index trend (δLAI) over 2001–2018.**

Areas with statistically significant trends are masked by the black dots (Mann-Kendall test, $P < 0.05$).

Source data are provided as a Source Data file.

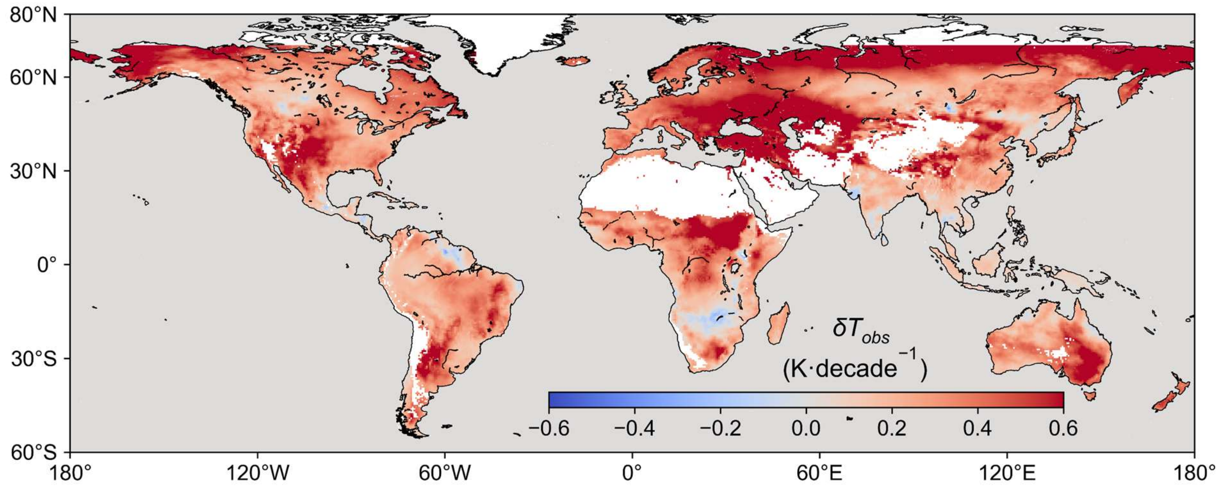

Supplementary Figure 9. **Global map of annual air temperature trend (δT_{obs}) over 1991–2018.**

Source data are provided as a Source Data file.

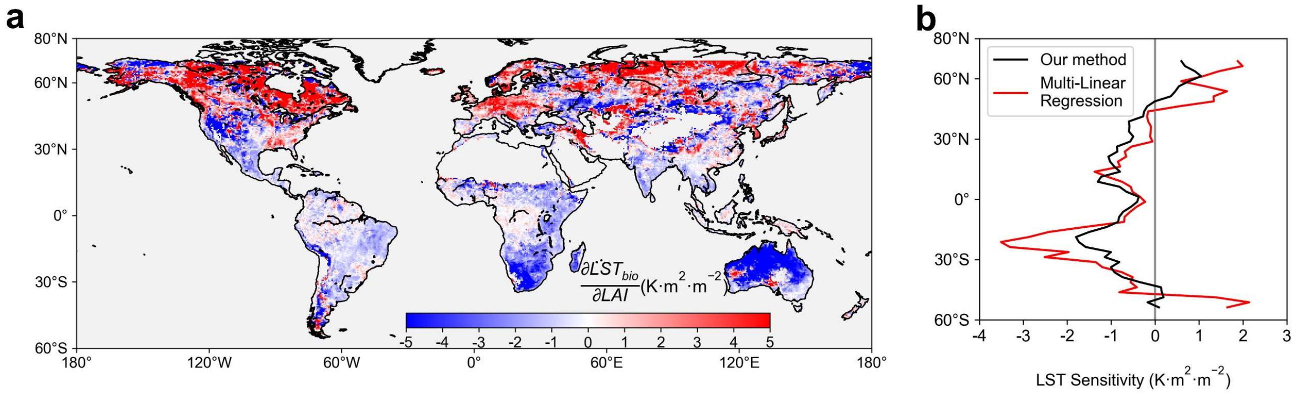

Supplementary Figure 10. **Comparison of annual biophysical sensitivity of land surface temperature to leaf area index derived from multi-linear regression method ($\boldsymbol{\frac{\partial LST_{bio}}{\partial LAI}}$) and our method ($\boldsymbol{\frac{dLST_{bio}}{dLAI}}$). a** Spatial map of $\frac{\partial LST_{bio}}{\partial LAI}$. **b** Comparison of latitudinal patterns between $\frac{\partial LST_{bio}}{\partial LAI}$ and $\frac{dLST_{bio}}{dLAI}$ (Fig. 1a). Source data are provided as a Source Data file.

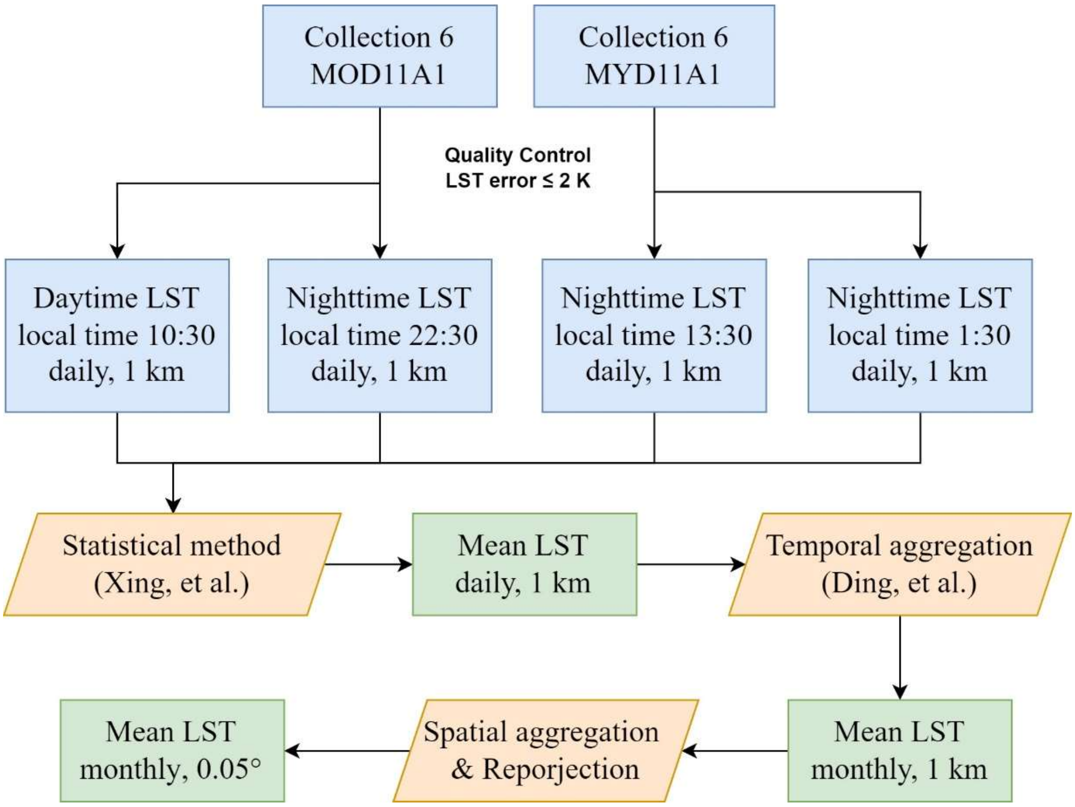

Supplementary Figure 11. **Flow chart for the monthly land surface temperature data production.**

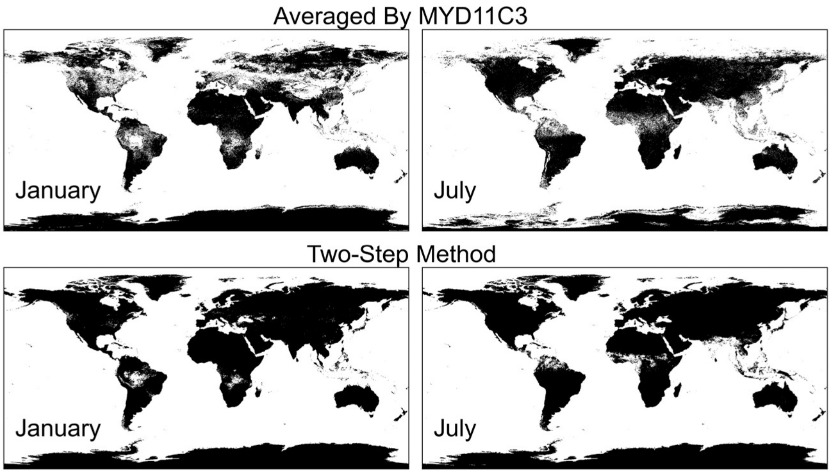

Supplementary Figure 12. **The comparison of the spatial coverage of monthly land surface temperature (LST) data generated from different strategies.** Spatial coverage of monthly LST by averaging MYD11C3 day and night observations in **a** January, 2010 and **b** July, 2010. Spatial coverage of monthly LST by our two-step method in **c** January, 2010 and **d** July, 2010.

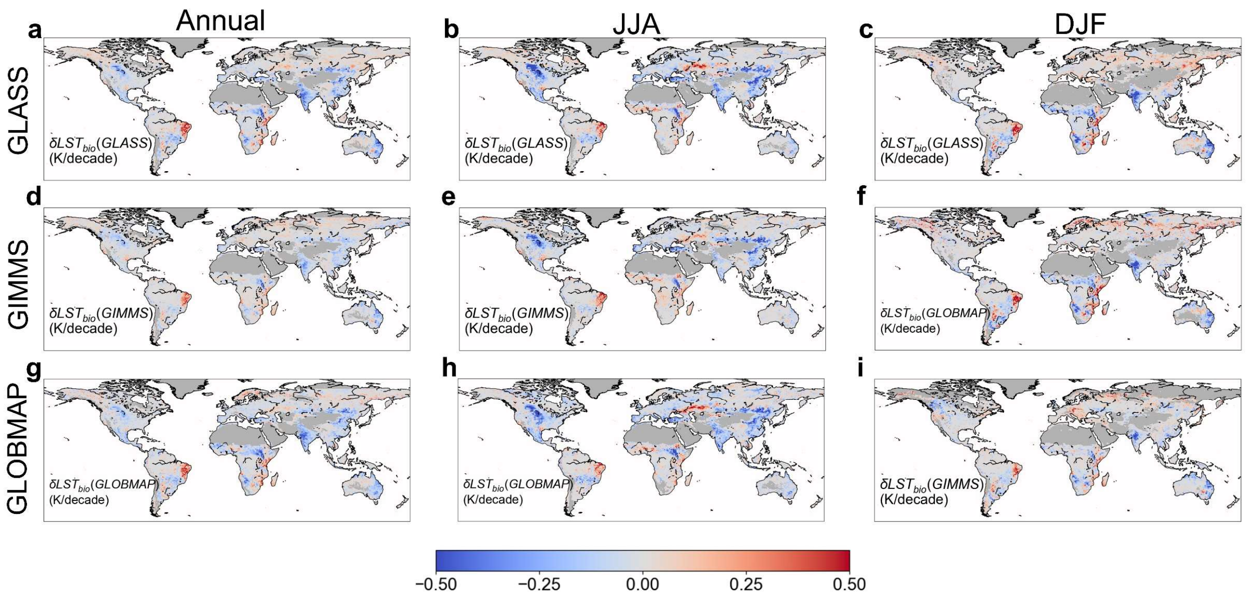

Supplementary Figure 13. **Spatial maps of greening induced land surface temperature trend (δLST_{bio}) estimated by GLASS, GIMMS and GLOBMAP leaf area index datasets.** **a** Annual mean, **b** JJA (June to August) mean and **c** DJF (December to February) mean of δLST_{bio} from GLASS. **d** to **f**, same as **a** to **c**, but for GIMSS δLST_{bio}. **g** to **i**, same as **a** to **c**, but for GLOBMAP δLST_{bio}. Source data are provided as a Source Data file.

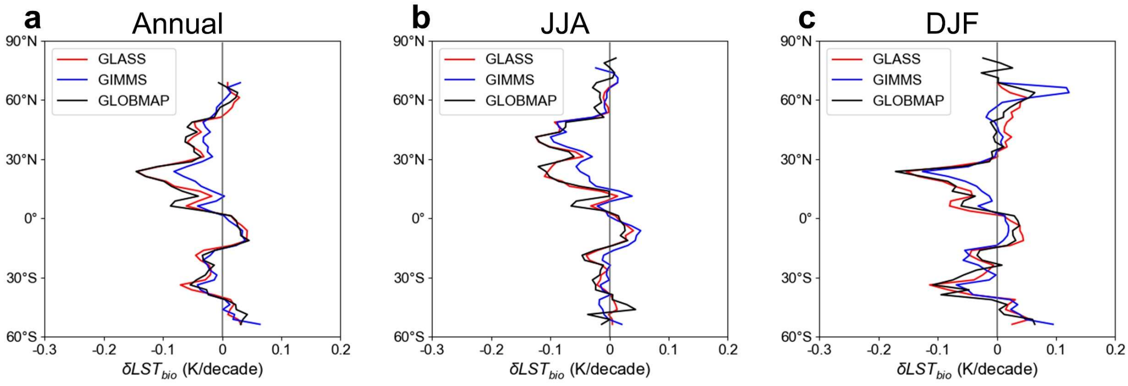

Supplementary Figure 14. **Latitudinal patterns of greening induced land surface temperature trend (δLST_{bio}) estimated by GLASS, GIMMS and GLOBMAP leaf area index (LAI) datasets.** Latitudinal patterns of **a** annual mean, **b** JJA (June to August) mean, and **c** DJF (December to February) mean of δLST_{bio} estimated by three LAI products. Source data are provided as a Source Data file.

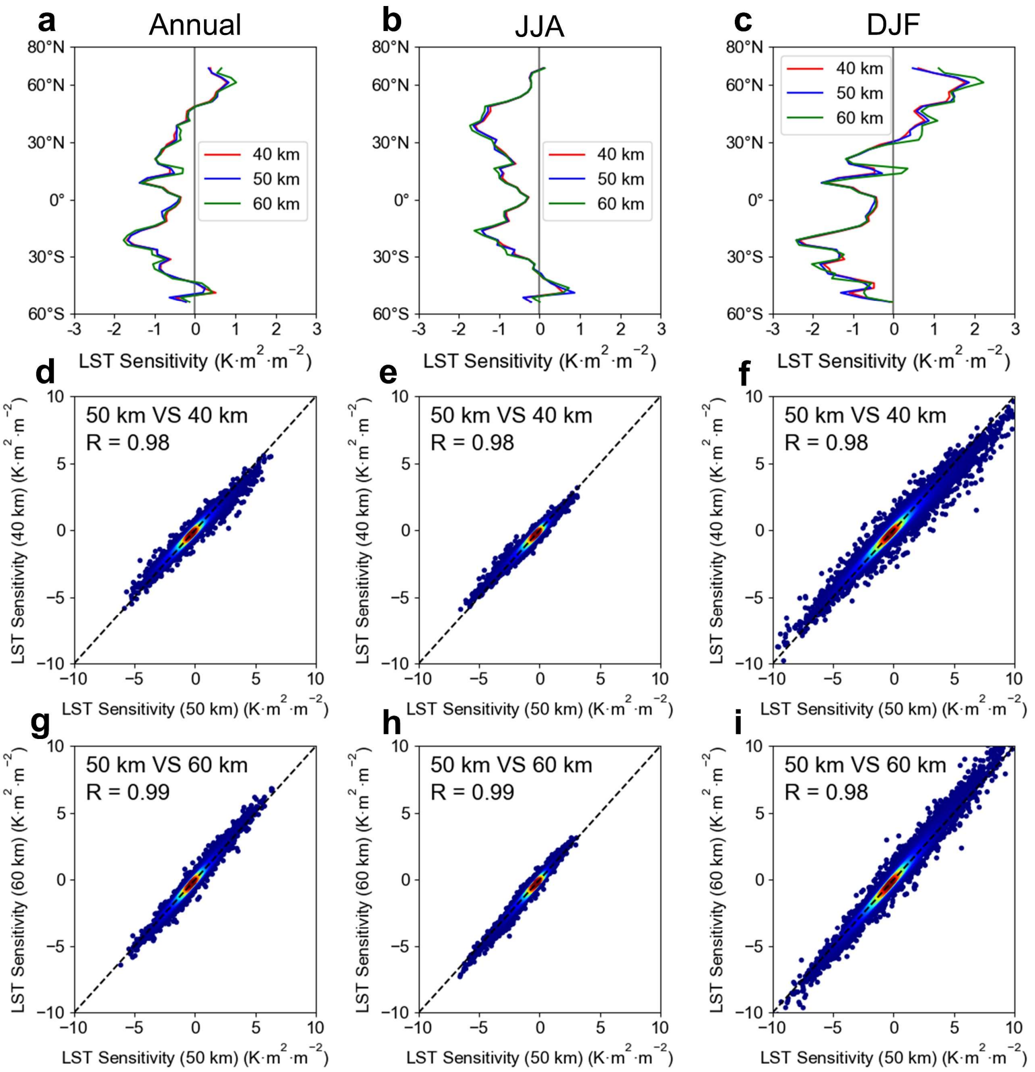

Supplementary Figure 15. **Comparison of land surface temperature (LST) sensitivity with different window sizes (40 km, 50 km, 60 km).** **a** to **c** Latitudinal patterns of LST sensitivity for annual mean, JJA (June to August) mean, and DJF (December to February) mean, respectively. **d** to **f** Density scatter plots between LST sensitivities derived from 50 km and 40 km for annual mean, JJA mean and DJF mean, respectively, respectively. **g** to **i** Same as **d** to **f**, but for the comparison between window sizes of 50 km and 60 km. Source data are provided as a Source Data file.

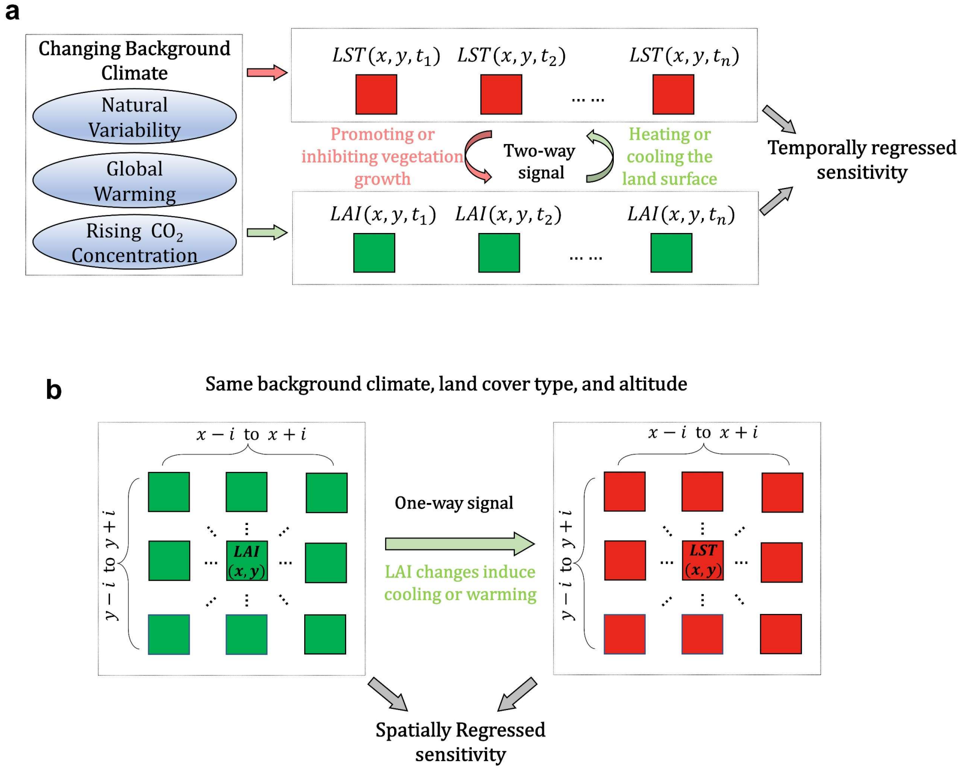

Supplementary Figure 16. **Schematic representation of the relationship between leaf area index (LAI) and land surface temperature (LST) from the temporal and spatial regression methods.** **a** Temporal regression. **b** Spatial regression.

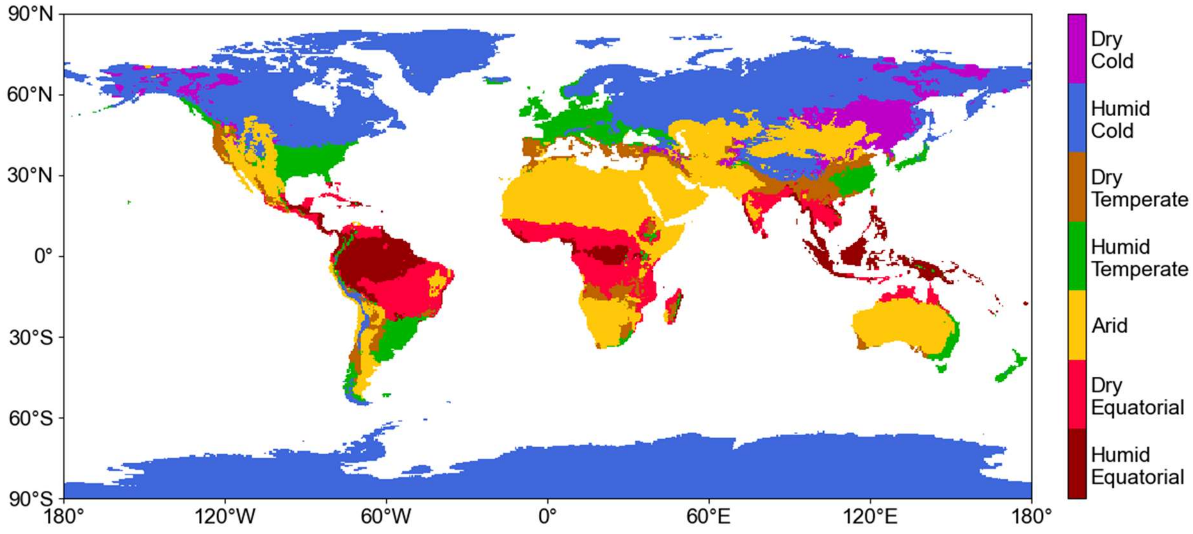

Supplementary Figure 17. **Seven major climate zones aggregated from the Köppen-Geiger climate classification map.**

Supplementary table

Supplementary Table 1: Broad vegetation type classification based on the International Geosphere-Biosphere Programme (IGBP) classification scheme[7]

Broad Vegetation Type	IGBP Type	IGBP Code
Forest	evergreen needleleaf forests (ENF)	1
	evergreen broadleaf forests (EBF)	2
	deciduous needleleaf forests (DNF)	3
	deciduous broadleaf forests (DBF)	4
	mixed forests (MF),	5
Other Woody Vegetation (OWV)	closed shrublands (CSH)	6
	open shrublands (OSH)	7
	woody savannas (WSA)	8
Grassland	savannas (SAV)	9
	grasslands (GRA)	10
Cropland	croplands (CRO)	12
	cropland-natural vegetation mosaic (CVM)	14

References

1. Xu, R. *et al.* Contrasting impacts of forests on cloud cover based on satellite observations. *Nat. Commun.* **13**, 670 (2022).

2. Zeng, Z. *et al.* Climate mitigation from vegetation biophysical feedbacks during the past three decades. *Nat. Clim. Chang.* **7**, 432–436 (2017).

3. Forzieri, G. *et al.* Increased control of vegetation on global terrestrial energy fluxes. *Nat. Clim. Chang.* **10**, 356–362 (2020).

4. Forzieri, G., Alkama, R., Miralles, D. G. & Cescatti, A. Satellites reveal contrasting responses of regional climate to the widespread greening of Earth. *Science (80-.).* **356**, 1180–1184 (2017).

5. Li, Y., Zeng, Z., Huang, L., Lian, X. & Piao, S. Comment on "Satellites reveal contrasting responses of regional climate to the widespread greening of Earth". *Science (80-.).* **360**, 1–3 (2018).

6. Jiang, C. *et al.* Inconsistencies of interannual variability and trends in long-term satellite leaf area index products. *Glob. Chang. Biol.* **23**, 4133–4146 (2017).

7. Chen, C. *et al.* Biophysical impacts of Earth greening largely controlled by aerodynamic resistance. *Sci. Adv.* **6**, 1–10 (2020).
